# Predictive Value of Gene Databases in Discovering New Biomarkers and New Therapeutic Targets in Lung Cancer

**DOI:** 10.3390/medicina59030547

**Published:** 2023-03-10

**Authors:** Mengfeng Liu, Xiran Yu, Changfa Qu, Shidong Xu

**Affiliations:** Department of Thoracic Surgery, Harbin Medical University Cancer Hospital, Harbin 150081, China

**Keywords:** lung cancer, immune cell infiltration, weighted gene co-expression, network analysis, protein–protein interaction networks, biomarkers

## Abstract

*Background and Objectives*: The molecular mechanisms of lung cancer are still unclear. Investigation of immune cell infiltration (ICI) and the hub gene will facilitate the identification of specific biomarkers. *Materials and Methods*: Key modules of ICI and immune cell-associated differential genes, as well as ICI profiles, were identified using lung cancer microarray data from the single sample gene set enrichment analysis (ssGSEA) and weighted gene co-expression network analysis (WGCNA) in the gene expression omnibus (GEO) database. Protein–protein interaction networks were used to identify hub genes. The receiver operating characteristic (ROC) curve was used to assess the diagnostic significance of the hub genes, and survival analysis was performed using gene expression profiling interactive analysis (GEPIA). *Results*: Significant changes in ICI were found in lung cancer tissues versus adjacent normal tissues. WGCNA results showed the highest correlation of yellow and blue modules with ICI. Protein–protein interaction networks identified four hub genes, namely CENPF, AURKA, PBK, and CCNB1. The lung adenocarcinoma patients in the low hub gene expression group showed higher overall survival and longer median survival than the high expression group. They were associated with a decreased risk of lung cancer in patients, indicating their potential role as cancer suppressor genes and potential targets for future therapeutic development. *Conclusions*: CENPF, AURKA, PBK, and CCNB1 show great potential as biomarkers and immunotherapeutic targets specific to lung cancer. Lung cancer patients’ prognoses are often foreseen using matched prognostic models, and genes CENPF, AURKA, PBK, and CCNB1 in lung cancer may serve as therapeutic targets, which require further investigations.

## 1. Introduction

Lung cancer is a malignancy with the highest prevalence and mortality rates worldwide [1]. More than half of lung cancer patients had metastases by the time of diagnosis. The lack of accurate biomarkers for early tumor diagnosis, as well as limited preclinical models, have hampered successful lung cancer treatment. To prevent the onset and progression of lung cancer, molecular identification is required for basic and clinical research and the discovery of novel and effective lung cancer prognostic markers. The Global Cancer Statistics 2020 report shows that the number of new lung cancer cases reached 2.2 million and that lung cancer is the malignancy with the highest incidence in male patients [1,2]. The development of lung cancer involves various genes and pathways, and the exact molecular mechanisms of its development and prognosis require in-depth research [3].

Bioinformatics is used to analyze genomic DNA sequence information, obtain information about protein-coding regions, perform protein spatial structure simulation and prediction, and then develop the necessary drug design based on the function of a specific protein. Genome informatics, protein spatial structure simulation, and drug design constitute the three major components of bioinformatics. With the development of the era of big biological data, a large amount of biological data has been accumulated to facilitate the construction of predictive models by bioinformatics to identify valid biomarkers. Identification of new lung cancer markers and therapeutic targets will provide insights into the diagnosis of lung cancer and improve patients’ quality of life. Messenger ribonucleic acid (mRNA) expression datasets have been used for biological purposes, which contributes to analyzing multigene expression profiles [4,5].

With the continuous progress of microfabrication technology, high-throughput immunoassay methods based on microarray technology have shown great potential for development. Microarray analysis of cancer has been widely adopted to identify genes and pathways of cancer to reveal the mechanisms of tumorigenesis. Microarray technology is a powerful technique for high-throughput parallel detection of proteins and other biomolecules, which is essentially a parallel analysis of a large amount of biological information on the same chip, allowing some traditional biological analysis to be done with as few reagents and at the highest possible speed in a minimum of space [6,7].

Tumor immune microenvironment (TIME) is the surrounding environment for tumor cells to survive, which is composed of blood vessels, immune cells, fibroblasts, and other cells and extracellular matrix [8,9]. Tumor cells can affect the surrounding microenvironment by expressing different ligands and receptors and secreting corresponding effector molecules, thus promoting tumor invasion and metastasis. Immune cells can also inhibit tumor growth through the above process [10]. TIME is an important place for tumor cells and immune cells to contend with each other, and the characteristics of TIME also determine the effectiveness of immunotherapy [9]. With the in-depth research in the field of tumor immunotherapeutics, tumor immune microenvironment has captured great attention in lung cancer treatment, and the superior efficacy of immune sentinel inhibitors represented by PD-1 and its ligand PD-L1 inhibitors has deepened the study of tumor immune microenvironment. Here, differentially expressed genes (DEGs) from three different datasets were integrated to minimize the false discovery rate (FDR), and bioinformatics analysis processes were performed to understand the immune cell infiltration (ICI) patterns and immune cell-related hub genes in lung cancer tissues.

## 2. Materials and Methods

### 2.1. Data Collection and Processing

Datasets were selected from the Gene Expression Omnibus (GEO). The strategy was to search (‘lung cancer’ [MeSH] [All Fields] normal) AND (‘Homo sapiens’ [Organism] AND coding RNA profiling by array’ [Filter]). Microarray datasets GSE27262, GSE18842, and GSE19804 including lung cancer samples and controls were collected from the gene expression omnibus (GEO) database (Table 1). Each dataset was preprocessed by (1) removing probes that did not correspond to Entrez GeneIDs; (2) retaining the probe with the largest expression in the probe set for one gene corresponding to multiple Entrez GeneIDs; (3) performing log2 transformation and normalization on each of the three microarray datasets; and (4) combining the three microarray datasets and performing de-batching effects. The batching effect is a common characteristic that arises in experimental results that causes data deviation from accuracy. It is mainly due to non-biological factors, such as sample collection time, collection organization, and sequencing platform, which may produce separate batches automatically, influencing the true data. As a result, the batching impact should be examined and eliminated before analysis. When integrating three separate GEO microarray data sets, the SVA package in R and the Combat function were used to manage the batching effect.

### 2.2. Identification of Differential Genes

The limma package (R language) [11] was employed to identify differential genes in cancerous tissues and adjacent normal tissues in three microarray datasets with a set threshold of |logFC| > 1 and adj. *p* value < 0.05.

### 2.3. Prediction of ICI Using Single-Sample Gene Set Enrichment Analysis (ssGSEA)

First, a set of genes acquired from Charoentong’s study was used to mark immune cell types [12,13], and ssGSEA was performed to derive differences in ICI between lung cancer tissues and adjacent normal tissues [14]. The ICI of the samples was predicted as an external trait of the sample using ssGSEA, which was used for the following analyses.

### 2.4. Establishment of Weighted Gene Co-Expression Network Analysis (WGCNA)

Identification of co-expression modules was performed using WGCNA with an R package [15]. Sample clustering was performed with the hclust function. When the scale-free topology fit index R2 reached 0.9, the optimal soft threshold was determined by the pickSoftThreshold function to meet the scale-free property of the biological network. A dynamic hybridization cleavage method was employed to identify genes with similar expression patterns as different modules, with a minimum number of module genes at 30 [16,17].

### 2.5. Identification of Immune Cell-Associated Differential Genes

Two WGCNA modules that linked most significantly with immune infiltration predicted by the ssGSEA were chosen to demonstrate the connection of co-expressed gene modules with ICI in lung cancer patients. To discover immune cell-associated differential genes, the target gene modules were intersected with differential genes.

### 2.6. Functional Enrichment and Pathway Analysis of Immune Cell-Associated DEGs

The enrichGO and enrichKEGG functions of the ClusterProfiler package in Bioconductor (http://bioconductor.org/packages/release/bioc/html/clusterProfer.html, accessed on 15 October 2022) were adopted for immune GO and KEGG enrichment analysis of cell-associated differential genes. With human genome as the background reference, a cut-off value of *p* < 0.05 was used to determine the GO and KEGG enrichment analysis of immune cell-associated differential genes. Finally, enrichment analysis results were displayed using the bar graphs in the ClusterProfiler package.

### 2.7. Protein-Protein Interaction (PPI) Network and Hub Gene Identification

The STRING database (http://string-db.org, accessed on 17 October 2022) used core factors as query proteins to evaluate PPI in functional protein association networks [18]. The PPI network of immune cell-associated differential genes was established on the STRING database. Hub genes in gene networks were recognized by the Cytohubba plugin in Cytoscape [19]. Despite that the close association of PPI network with mRNA expressions, following differential analysis was to screen for hub genes using PPI networks, which had no effect on the results.

### 2.8. Hub Gene Expression and Its Diagnostic Performance

The mean values of hub gene expression in lung cancer tissues and the adjacent normal tissues were compared by the Wilcoxon rank-sum test. The ROC curve of hub genes against lung cancer was plotted with the pROC package in R, and the AUC was used to evaluate the diagnostic value of hub genes.

### 2.9. Correlation and Survival Analysis of Hub Genes with Tumor-Infiltrating Immune Cells

The TIMER database was adopted to assess the relationship between hub gene expression levels and infiltrating immune cell components in lung cancer [20]. Survival analysis was conducted using gene expression profiling interactive analysis (GEPIA) to explore the association of hub gene expression levels with lung cancer prognosis [21]. The GEPIA database allows the use of the TCGA dataset to assess gene survival outcomes.

## 3. Results

The purpose of this study is to investigate, evaluate, and summarize the evidence of weighted gene co-expression network analysis in the tissue of lung cancer patients, so as to determine useful biomarkers, new therapeutic targets, and prognosis evaluation methodologies in the therapy of lung cancer.

In this study, we selected three GEO datasets (GSE27262, GSE18842 and GSE19804) as our objects. GSE27262 included RNA chip data from 25 patients with lung adenocarcinoma and adjacent tissues. GSE18842 included RNA chip data from cancer tissues of 46 patients with non-small cell lung cancer (NSCLC) and 45 normal tissues. GSE19804 included RNA chip data from cancer and adjacent tissues of 60 non-smoking female patients with lung cancer.

### 3.1. Research Flow Chart

**Figure 1:**
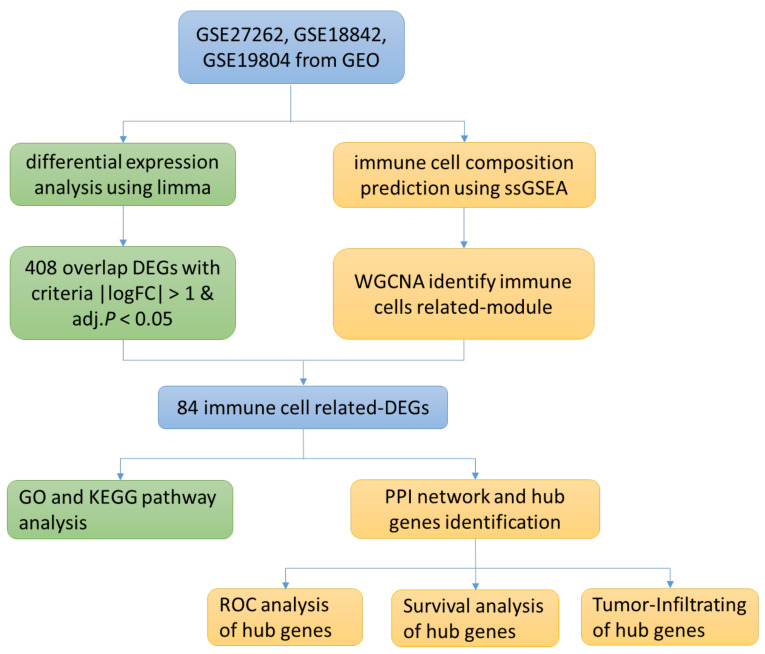
Research Flow Chart.

The research flow chart of the present study is in Figure 1. GSE27262, GSE18842 and GSE19804 from GEO were selected. We used limma package and ssGSEA analysis to search for differential expression genes (DEGs) and immune-cell related genes, respectively, and used Venn diagrams to screen overlapping genes. GO and KEGG analysis were performed on the selected genes, and the protein interaction network was established. Finally, ROC curve and survival curve were drawn according to the clinical data provided by the database.

### 3.2. Differential Gene Identification

**Figure 2:**
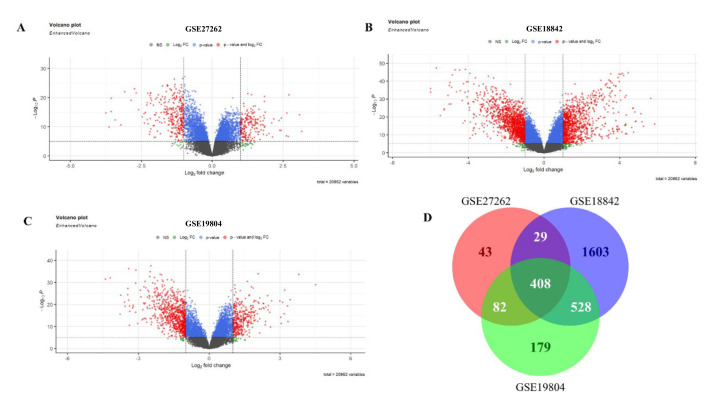
DEGs in the microarray dataset. (**A**): DEGs of GSE27262; (**B**): DEGs of GSE18842; (**C**): DEGs of GSE19804; (**D**): DEGs shared by 3 microarray datasets.

The limma package was adopted to identify differential genes in three microarray datasets, namely GSE27262, GSE18842, and GSE19804 (Table 1). We analyzed differential genes in three databases, and the results were presented as volcanic maps (Figure 2A–C). A total of 562 DEGs were obtained in GSE27262. A total of 2568 DEGs were obtained in GSE18842. In GSE19804, a total of 1197 DEGs were obtained. Venn diagrams were used to analyze the DEGs in the above three data sets. Finally, 408 overlapping DEGs were identified (Figure 2D).

### 3.3. Identification of Immune Cell-Related Module Genes by ssGSEA and WGCNA

**Figure 3:**
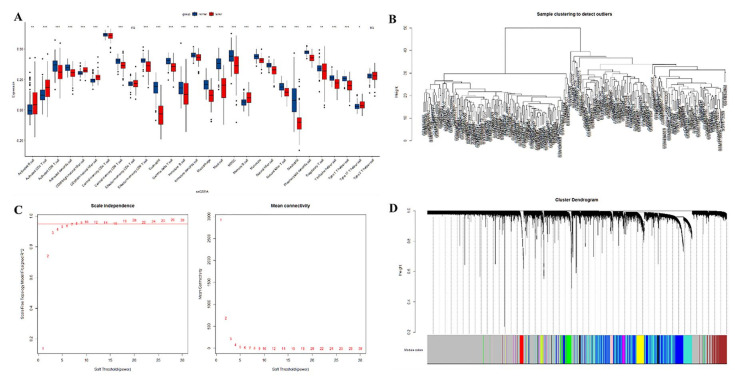
(**A**): Prediction of the distribution of immune infiltrating cells between lung cancer tissue and adjacent normal lung tissue by ssGSEA. The *x*-axis shows the different immune cells, and the *y*-axis shows the scores of the ssGSEA. * indicates *p* < 0.05, ** indicates *p* < 0.01, *** indicates *p* < 0.001, and **** indicates *p* < 0.0001. Differences were considered statistically significant at *p* < 0.05, and ns indicates that the differences are not statistically significant. (**B**): sample clustering; (**C**): selection of optimal soft thresholds for WGCNA; (**D**): tree diagram of the WGCNA module.

**Figure 4:**
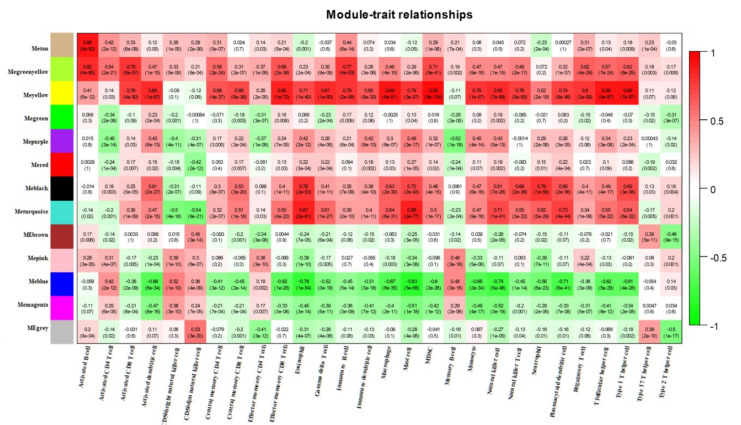
Heat map of the correlation between co-expression modules identified by WGCNA and immune infiltrating cells predicted by ssGSEA.

The difference in ICI between lung cancer tissue and adjacent normal tissue was assessed using ssGSEA. Between cancerous tissues and adjacent normal tissues, significant alterations were observed in cells such as activated B cell, activated CD4 T cell, and activated CD8 T cell, while the effector memory CD4 T cells and type 2 T helper cells were similar (Figure 3A). The three sets of microarray data were pooled and subjected to de-batching effects, and a total of 20,862 genes were included to establish a weighted gene co-expression network. Sample clustering was conducted by hierarchical clustering and no significant outliers were found (Figure 3B). The optimal soft threshold value of 9 was selected by the pickSoftThreshold function (Figure 3C,D). Thirteen co-expression modules were identified, and the association of ssGSEA-predicted ICI with the 13 co-expression modules was further explored. The results showed the closest association of yellow and blue modules with ICI (Figure 4).

### 3.4. Functional Enrichment Analysis of Immune Cell-Associated Differential Genes

**Figure 5:**
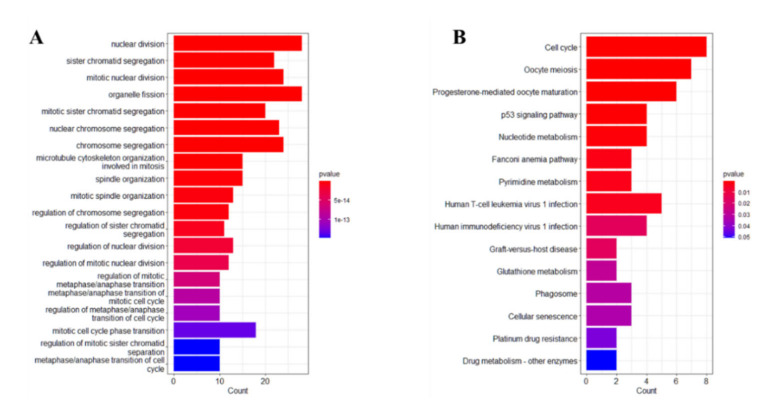
GO and KEGG enrichment analysis. (**A**): GO functional enrichment analysis; (**B**): KEGG functional enrichment analysis.

The yellow and blue gene modules were intersected with the differential genes to identify 86 immune cell-associated DEGs. The results of GO enrichment analysis and KEGG enrichment analysis for lung cancer immune cell-related DEGs are shown in Figure 5A,B.

GO enrichment analysis showed that the lung cancer immune cell-related DEGs were related to nuclear division, sister chromatid segregation, mitotic nuclear division, organelle fission, miotic sister chromatid segregation, chromosome segregation, microtubule cytoskeleton organization involved in mitosis, etc. KEGG functional enrichment analysis showed that lung cancer immune cell-related DEGs were related to the cell cycle, oocyte meiosis, progesterone-mediated oocyte maturation, p52 signaling pathway, nucleotide metabolism, Fanconi anemia pathway and so on.

### 3.5. Identification of Hub Genes and ROC Curve Analysis

**Figure 6:**
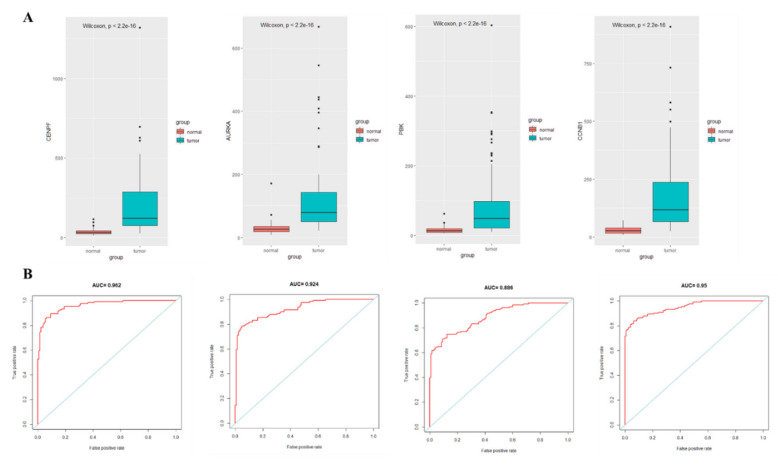
Expression and ROC curve analysis of hub gene in lung cancer tissue and adjacent normal lung tissue. (**A**): Hub gene expression; (**B**): ROC curve analysis of hub gene.

The PPI network was constructed by entering 86 immune cell-related differential genes in the STRING database, and the Cytohubba plug-in in Cytoscape identified four hub genes, namely Centromere Protein F (CENPF), Aurora kinase A (AURKA), PDZ-binding kinase (PBK), and Cyclin B1 (CCNB1). Moreover, high expression of the four hub genes was observed in lung cancer tissues with statistically significant differences (Figure 6A). The ROC curves demonstrated good diagnostic efficacy for lung cancer, with AUC values of 0.962, 0.924, 0.886, and 0.95 for the four hub genes CENPF, AURKA, PBK, and CCNB1, respectively (Figure 6B). The AUC of diagnosing model by four hub genes (BENBF, AURKA, PBK, CCNB1) was 0.974 (Figure 6B). The AUC of combined diagnosis is higher than that of single gene, which indicates that the four hub genes are highly effective in the diagnosis of lung cancer.

### 3.6. Correlation and Survival Analysis of Hub Gene and Tumor-Infiltrating Immune Cells

**Figure 7:**
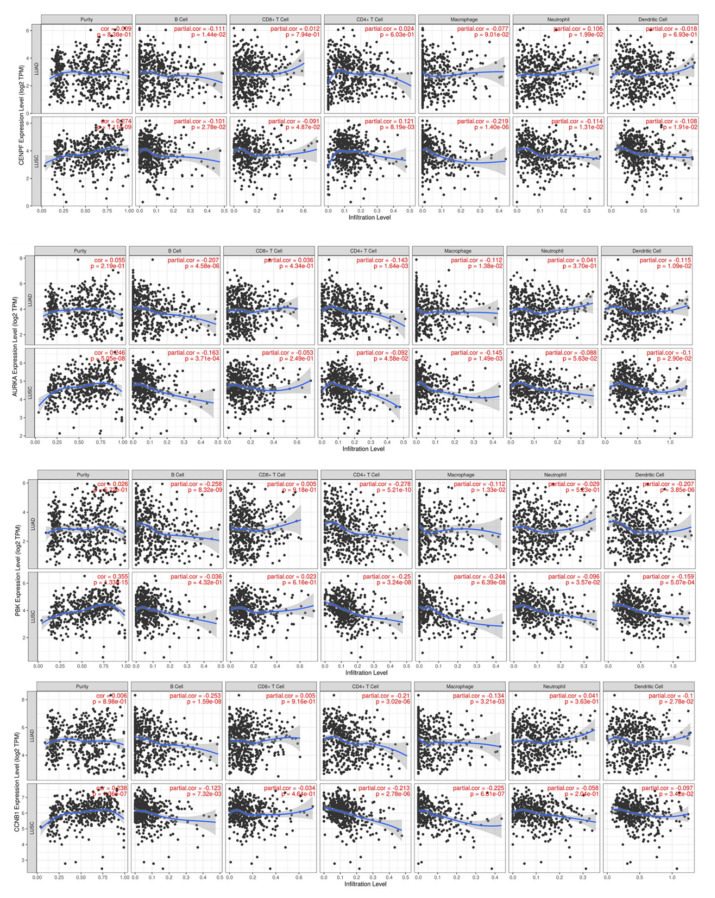
Correlation of hub genes with tumor-infiltrating immune cells.

In lung adenocarcinoma (LUAD), there was a correlation between the expression of CENPFand B cells and neutrophils infiltration, AURKA and B cells, CD4+ T cells, macrophages, and dendritic cells infiltration, PBK and B cells, CD4+ T cells, macrophages, and dendritic cells infiltration, and CCNB1 and B cells, CD4+ T cells, macrophages, and dendritic cells infiltration (*p* < 0.05) (Figure 7). In lung squamous carcinoma (LUSC), there was a correlation between the expression of CENPF and tumor purity, B cells, CD8+ T cells, CD4+ T cells, macrophages, neutrophils, and dendritic cells infiltration, AURKA and tumor purity, B cells, CD4+ T cells, macrophages, and dendritic cells infiltration, PBK and tumor purity, CD4+ T cells, macrophages, neutrophils, and dendritic cells infiltration, and CCNB1 and tumor purity, B cells, CD4+ T cells, macrophages, and dendritic cell infiltration (*p* < 0.05) (Figure 7).

**Figure 8:**
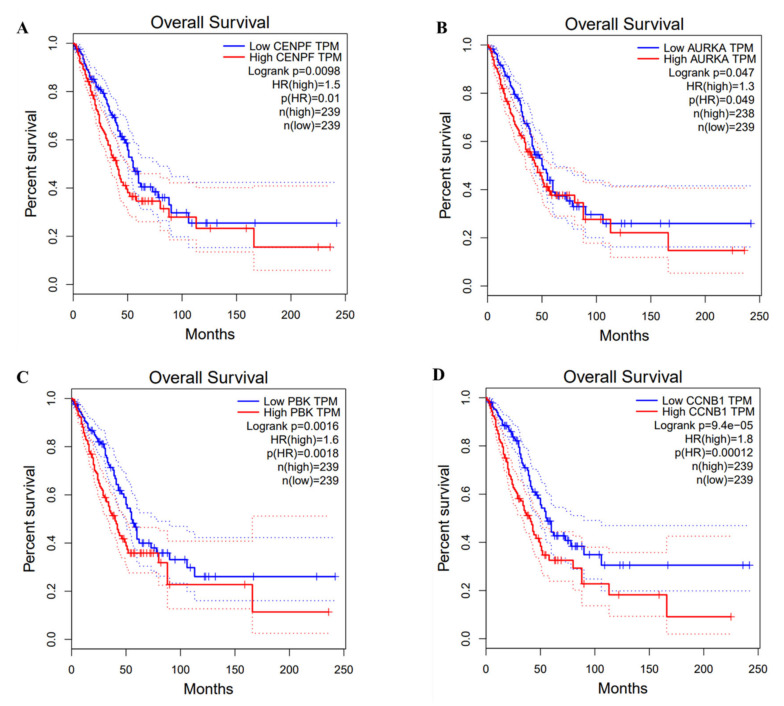
Survival analysis of hub genes and lung cancer.

The overall survival rate and median survival time of LUAD patients in the hub genes low expression group had higher overall survival rates and longer median survival of LUAD patients than the high expression group (*p* < 0.05) (Figure 8).

## 4. Discussion

Investigation of genetic biomarkers associated with ICI and disease progression in lung cancer contributes to improving diagnostic accuracy. Herein, various bioinformatics approaches were adopted to identify hub genes linked to ICI in lung cancer and their roles in lung cancer diagnosis and prognosis.

AURKA is a low episodic tumor susceptibility gene regulator and centrosome in the cell cycle [22,23]. AURKA has been well studied in multiple cancers, such as gastrointestinal, colorectal, breast, bladder, and lung cancers [24,25,26,27,28]. According to recent studies, higher AURKA expression demonstrated poor prediction outcomes for non-small cell lung cancer [29]. VX-680, hesperidin, AZD1152, and MLN8237 are AURKA inhibitors that have been proven to have anticancer therapeutic benefits [30]. Other studies [31,32,33] showed that AURKA could be useful as an immunotherapeutic target. Wang et al. [34] found that inhibition of AURKA to induce autophagy apoptosis could promote radiotherapy sensitivity in patients with non-small cell lung cancer. Santos et al. [32] showed that AURKA was related to the proliferation, invasion, and migration of tumor cells. When AURKA expression was inhibited, the proliferation and invasion of tumor cells were reduced, and apoptosis was more likely. In addition, Zhang et al. [33] found that AURKA was highly expressed in lung cancer patients and negatively correlated with the proportion of B lymphocytes and dendritic cells in tumors, which may be associated with poor prognosis of patients.

The expression of CCNB1 correlates with the development of cancer [35]. It has been found that CCNB1s are up-regulated in some tumor cells, such as lung cancer, and also participate in mitosis by creating a maturation complex that promotes the cellular G2/M transition and initiates mitotic progression. This gene product interacts with p34 (CDC2) to generate a maturation-promoting factor and regulates the G2/M transition phase [30]. In addition, CCNB1 is considered a potential diagnostic biomarker for rhabdomyosarcoma and estrogen receptor (ER)-positive breast cancer [36].

CENPF plays a key role in chromosome segregation processes [37]. An increasing amount of data reveals that CENPF overexpression is strongly linked to cancer development and prognosis [38,39,40,41,42,43]. High CENPF expression was found in lung tumors with FHIT and p53 inactivation, according to Andriani et al. [44]. Nonetheless, there has been little research on CENPF’s oncogenic significance in lung cancer. CENPF is also overexpressed in a variety of malignancies and is associated with aggressive tumor characteristics and prognosis [45,46,47].

PBK is a MAPKK-like protein kinase, and its high expression is associated with multiple aggressive cancer phenotypes [48], and PBK is considered a potential molecular target for tumor diagnosis and targeted therapy [49]. Currently, there is increasing body of evidence suggesting its key role in carcinogenesis and metastasis. Xu et al. demonstrated the association of overexpression of PBK in many tumors with poor survival outcomes [50]. The obtained hub genes had a good correlation with tumor purity as well as the ratio of the infiltrating immune cells. The hub genes after GEPIA database analysis were correlated with prognosis of LUAD patients, suggesting that the four hub genes show great potential to be biomarkers and immunotherapeutic targets specific to lung cancer.

The pathways focus on nuclear division, mitotic nuclear division, organelle fission, regulation of nuclear division, regulation of mitotic nuclear division, and cell cycle, indicating that when compared to nearby normal lung tissues, the key immune cell-associated differential genes in lung cancer tissues engaged in the pathway are connected to tumor incidence and progression. The cell cycle consists mostly of the cell cycle driving mechanism and the cell cycle regulating system. When the cell cycle regulating mechanism is disrupted, normal cell proliferation becomes uncontrollable, and cells convert into tumor cells. When the different stages of cell division and proliferation are affected by internal and external factors, it will also lead to abnormal differentiation and proliferation, resulting in tumorigenesis. Bioinformatics analysis confirmed that P53 is a target gene for some miRNAs in lung cancer cells. The results showed that miR-15a-5p knockdown led to a decrease in P53 in lung cancer cells, while miR-15a-5p overexpression led to an increase in P53 [51]. The oncoprotein P53 is one of the most studied oncogenes. p53 inhibits tumor formation and protects DNA from damage by inducing cell cycle arrest, DNA repair or apoptosis. Inhibition of P53 has been shown to be associated with drug resistance and poor prognosis in lung cancer patients, and increased P53 expression improves the sensitivity of lung cancer cells to cisplatin treatment [52]. As a result, P53 is a key molecule in the regulation of chemoresistance in lung cancer cells. Recent studies have shown that miRNAs such as miR-125b, miR-504, miR-25 and miR-30d could regulate the abundance and activity of P53 and negatively regulate P53 [53].

This study has the following limitations. First, this study was based on different data sets, among which there might be errors due to sequencing technology, chip platform, batch effect and data processing. Second, the present study tends to center on the signature’s four risk-genes in early expression study, while no additional functional or mechanical analysis was conducted. Finally, no lung cancer studies were conducted to substantiate the link between prognostic genes and shift. Future studies are required to provide more reliable data.

## 5. Conclusions

This study integrated three different GEO microarray datasets between lung cancer tissues and adjacent normal tissues using bioinformatics tools such as ssGSEA, WGCNA, and PPI to identify hub genes in these microarray datasets. The genes may involve in the oncogenesis of lung cancer. Hub genes may participate in regulating the tumor immune microenvironment. These findings contribute to the elucidation of the biological mechanisms of lung cancer and offer new biomarkers, and cENPF, AURKA, PBK, and CCNB1 show great potential as specific biomarkers and immunotherapeutic targets for lung cancer.

## Figures and Tables

**Table 1:** The GEO gene expression datasets description.

GEO	Platform	Tumor	Normal	DEGs
GSE27262	GPL570	25	25	562
GSE18842	GPL570	46	45	2568
GSE19804	GPL570	60	60	1197

## Data Availability

All data generated or analyzed during this study are included in this published article.
